# Essential metrics for assessing sex & gender integration in health research proposals involving human participants

**DOI:** 10.1371/journal.pone.0182812

**Published:** 2017-08-30

**Authors:** Suzanne Day, Robin Mason, Cara Tannenbaum, Paula A. Rochon

**Affiliations:** 1 Women’s Xchange, Women’s College Hospital, Toronto, Ontario, Canada; 2 Women’s College Research Institute, Women’s College Hospital, Toronto, Ontario, Canada; 3 Dalla Lana School of Public Health and Department of Psychiatry, University of Toronto, Toronto, Ontario, Canada; 4 Institute of Gender and Health, Canadian Institutes of Health Research, Montreal, Quebec, Canada; 5 Department of Medicine, University of Toronto, Toronto, Ontario, Canada; University of Oxford, UNITED KINGDOM

## Abstract

Integrating sex and gender in health research is essential to produce the best possible evidence to inform health care. Comprehensive integration of sex and gender requires considering these variables from the very beginning of the research process, starting at the proposal stage. To promote excellence in sex and gender integration, we have developed a set of metrics to assess the quality of sex and gender integration in research proposals. These metrics are designed to assist both researchers in developing proposals and reviewers in making funding decisions. We developed this tool through an iterative three-stage method involving 1) review of existing sex and gender integration resources and initial metrics design, 2) expert review and feedback via anonymous online survey (Likert scale and open-ended questions), and 3) analysis of feedback data and collective revision of the metrics. We received feedback on the initial metrics draft from 20 reviewers with expertise in conducting sex- and/or gender-based health research. The majority of reviewers responded positively to questions regarding the utility, clarity and completeness of the metrics, and all reviewers provided responses to open-ended questions about suggestions for improvements. Coding and analysis of responses identified three domains for improvement: clarifying terminology, refining content, and broadening applicability. Based on this analysis we revised the metrics into the *Essential Metrics for Assessing Sex and Gender Integration in Health Research Proposals Involving Human Participants*, which outlines criteria for excellence within each proposal component and provides illustrative examples to support implementation. By enhancing the quality of sex and gender integration in proposals, the metrics will help to foster comprehensive, meaningful integration of sex and gender throughout each stage of the research process, resulting in better quality evidence to inform health care for all.

## Introduction

Both sex and gender are fundamental to consider in the design of health research in order to produce the most complete and accurate evidence to inform health care and improve patient outcomes [1–3]. The terms ‘sex’ and ‘gender’ refer to two distinct but interrelated factors that shape health: sex encompasses a set of biological attributes such as chromosomes, gene expression, and anatomy, while gender refers to socioculturally-constructed roles, behaviours, norms, identities, and power relations [4]. Despite their importance, the integration of sex and gender in health research has yet to be consistently adopted as a standard research and reporting practice [5–9]. This indicates a need for tools and strategies to help researchers enhance the integration of sex and gender in their work.

Some recently developed tools can help guide sex and gender inclusion in the reporting of research findings in scientific publications, such as the Sex and Gender Equity in Research (SAGER) guidelines [10], and criteria for inclusion of sex and gender in Cochrane systematic reviews [11]. To conduct robust analyses, sex and gender need to be considered from the outset and integrated throughout the entire study [12], from research questions through methods, analysis plan, and dissemination strategy. Amidst growing interest on the part of federal health research funding agencies to include both sex and gender as a condition of funding [13, 14], researchers and reviewers would benefit from a way to assess the strength of that integration in proposals.

Some tools have been developed for this purpose. For example, the National Institutes of Health Research provides a decision tree for reviewers assessing the integration of sex as a biological variable in proposals [15]. This decision tree is limited to assessing only the integration of sex and not gender in the proposed research design and planned analyses. GENDER-NET, a European Research Area Network focusing on the integration of sex and gender analysis, has developed a set of guidelines and checklists for Integrating Gender Analysis in Research (IGAR) for both research applicants [16] and application reviewers [17]. These checklists do not include an assessment scale, and thus can only record the presence or absence of sex/gender considerations. Similarly, Tomás et al.’s 10-item questionnaire, Gender Perspectives in Health Research [18], permits only ‘yes’ or ‘no’ responses to a series of questions on sex and gender integration and thus cannot assess integration quality. Additionally, as the authors themselves note [18], reviewers require training in a gender perspective in order to appropriately use the questionnaire, posing a barrier to its widespread uptake.

With the objective of addressing the need for a comprehensive tool to assess the quality of sex and gender integration in health research proposals, we developed the *Essential Metrics for Assessing Sex & Gender Integration in Health Research Proposals Involving Human Participants*. This tool will be useful to both researchers preparing proposals and reviewers assessing proposals for funding opportunities. In this paper we detail the methods of our metrics development study and present the resulting tool.

## Methods

An iterative 3-stage method was used to develop the metrics. The Research Ethics Board at Women’s College Hospital reviewed and provided ethics exemption for this study.

### Review of existing tools

We began by compiling existing guidelines and tools for sex and gender integration. A selected bibliography was developed of 19 sex and gender integration resources that our group had previously used in our capacity as a women’s health research knowledge translation and exchange centre [1, 18–35]. This initial bibliography was then presented during a 2-day meeting of our research team. At this meeting, discussions focused on definitions of sex and gender, contexts for promoting sex and gender integration, and assessing the existing resources for sex- and gender-based analysis, adding to the initial bibliography based on the collective knowledge of the research team. The research process was mapped from start to finish to identify points where sex and gender should be integrated. Drawing upon five key publications unanimously agreed upon at our meeting to be the most comprehensive instructional tools available at the time [14, 23, 29, 36, 37], we developed an initial set of metrics designed to consider sex and gender at six pivotal points in the research process: literature review and research question development, research design and methods, data analysis, data reporting, knowledge translation planning, and patient engagement. In line with recent recommendations for translating sex- and gender-inclusive policy into practice [38], a descriptive assessment rather than a numeric scoring system was devised. Five assessment categories were provided for each proposal component, scaled from “poor” to “outstanding”.

### Expert review and feedback

Following methods used to develop sex and gender reporting guidelines [10, 11], we used purposive sampling to recruit Canadian and international health researchers with expertise in conducting sex- and/or gender-based analysis to review and provide feedback on the draft metrics. Potential reviewers were identified through a variety of means, including: 1) membership with the International Society for Gender Medicine and its affiliated national societies in Austria, the USA, Germany, Israel, Italy, Japan, and Sweden [39]; 2) involvement in developing existing tools for sex and gender integration in health research, such as those used to develop the metrics [14, 23, 29, 36, 37]; and 3) known associations among our team members’ research networks. Eligibility criteria included possession of an advanced degree (PhD or MD) and authorship of instructional resources and/or peer-reviewed journal articles on the integration of sex and gender considerations in health research.

A total of 55 individuals with expertise in sex- and gender-based health research were invited to review and provide feedback on the metrics, including 36 Canadian researchers and 19 international researchers. To encourage participation in the review process, invitees received a personalized email [40] with a link to a questionnaire hosted on a free online survey platform (Fluidsurvey.com).

Survey questions were developed based on a consensus discussion among our team as to what kinds of feedback would be most helpful for comprehensively revising the metrics. From this discussion we devised a survey composed of a mix of Likert scale responses and open-ended questions. The Likert scale questions solicited reviewers’ opinions on whether the metrics were clear, useful and complete. The open-ended questions allowed participants an opportunity to offer specific recommendations for improvement by asking “What additional questions should we include?”, “How could the metrics be made clearer and easier to use?” and “What additional comments/feedback do you have about the metrics?”. We also collected information about reviewers’ disciplinary backgrounds to better contextualize the recommendations and to verify that feedback was gathered from differing perspectives and a range of expertise. All responses were collected anonymously in order to reduce the risk of social desirability bias influencing reviewers’ reactions to the metrics.

### Qualitative analysis of feedback and revision

Expert reviewers’ responses were analyzed and used to inform subsequent revisions of the metrics. All survey responses were read and independently coded by two of the authors (SD and RM) using open codes to identify emergent themes. Open codes were compared and through discussion, consensus was reached on their synthesis into broader categories [41]. The categories reflect the expert reviewers’ primary recommendations, indicating the main changes that needed to be made to the tool. Over the course of several meetings, SD, RM, and PR discussed the categories and primary recommendations, and drafted and reviewed subsequent iterations to finalize the metrics.

### Patient involvement

No patients were involved in the design of this study, and there are no plans to disseminate the findings to patient groups.

## Results

### Expert review and feedback

**Fig 1:**
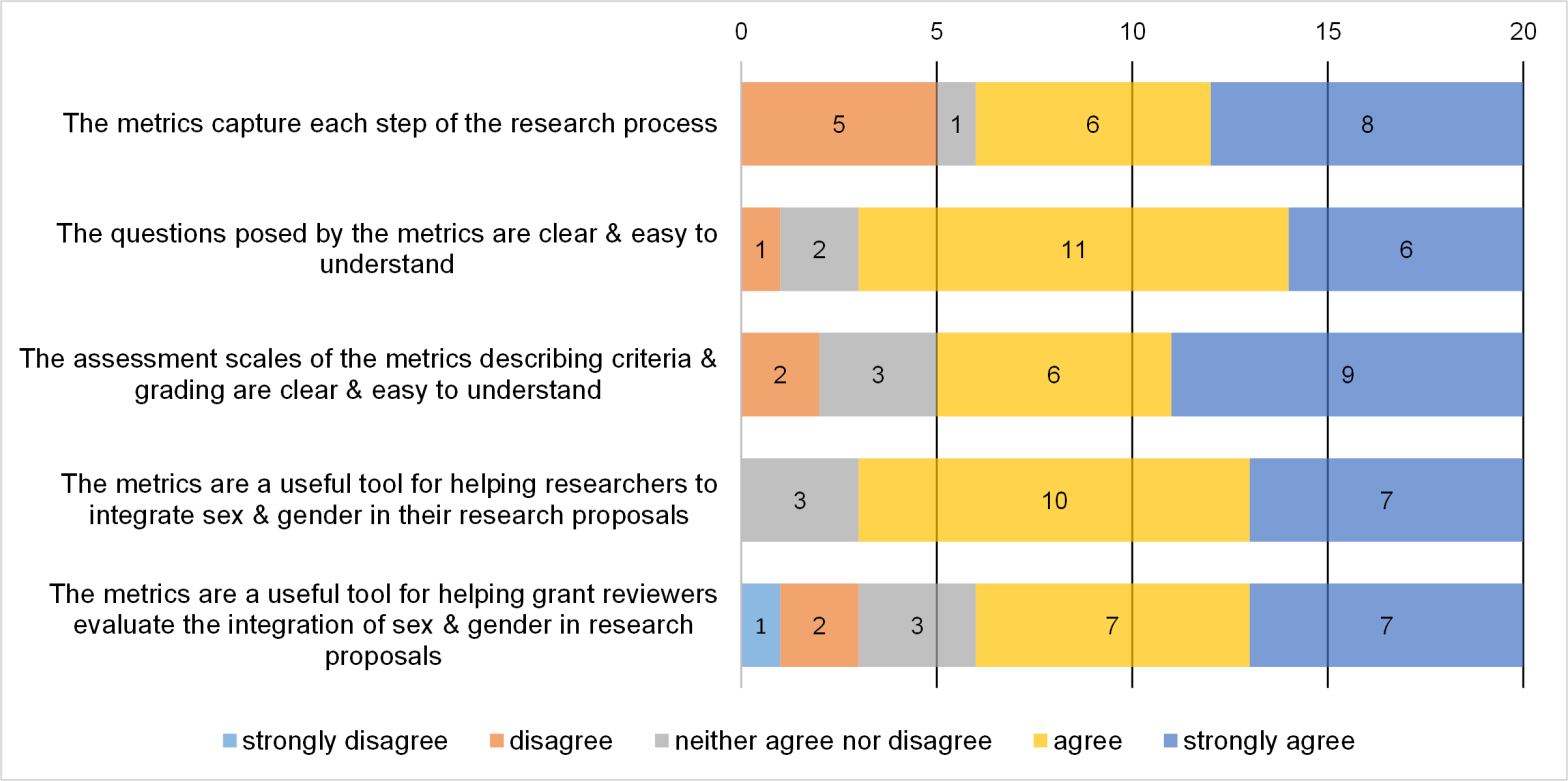
Reviewer responses to Likert scale questions on the metrics. Fig 1 shows the number of respondents that indicated strongly disagree, disagree, neither agree nor disagree, agree, and strongly disagree in response to each of the 5 survey questions.

We received a total of 20 completed online survey responses from 10 Canadian and 10 international (non-Canadian) experts in sex- and gender-based health research. Reviewers represented diverse disciplinary backgrounds, including epidemiology, physiology, pharmacology, neuroscience, ergonomics, social sciences, the humanities, medicine, and nursing. In their own research, 11 respondents indicated that they use both qualitative and quantitative methods, while 8 reported using quantitative methods exclusively and 1 reported using qualitative methods exclusively. In Likert scale responses, most reviewers indicated that they agreed or strongly agreed with questions on the clarity, completeness and utility of the metrics (Fig 1).

### Analysis of feedback and revision

All reviewers responded to the three open-ended survey questions. Open coding of this data yielded three broad categories of recommendations, indicating a need for: 1) clarifying terminology, 2) refining content, and 3) broadening applicability. Regarding terminology, reviewers noted some words were too vague, confusing or required further explanation. For example, use of the phrase “diverse populations” was confusing as there are many ways to address diversity (e.g. racial diversity, diverse socio-economic backgrounds, etc.). A fifth assessment level, “outstanding”, created challenges due to difficulties distinguishing between “outstanding” and “excellent”. In terms of content, additional points where sex and gender integration should be evaluated (such as in planning for retention of participants) were proposed. Finally, recommendations for broadening applicability of the metrics to a wider range of study types and research methodologies, such as single-sex studies and qualitative designs, were supplied by several reviewers.

Based on this analysis of reviewers’ recommendations, we revised the metrics in a series of iterations. Terms flagged by reviewers as being unclear were removed or replaced with clearer language. The assessment scale was reduced to 4 levels, and examples were embedded within the tool to help assess each specific measure as well as to illustrate how the metrics can be applied to different kinds of studies. Our analysis of reviewers’ feedback also indicated the need for a contextualizing set of instructions in order to interpret and effectively apply the metrics.

### Instructions for metrics use

The metrics are to be used by health researchers as they prepare their funding proposals, as well as by health research funding bodies as they review proposals submitted for funding. In applying the metrics, researchers and reviewers should keep four key considerations in mind:

- First, researchers and reviewers should be aware of the difference between the concepts of sex and gender, with sex referring to biological factors and gender referring to sociocultural factors [14].
- Second, investigating both sex *and* gender may not be appropriate for all study analyses. The relevance of sex and gender will depend upon whether the study proposes to investigate biological or sociocultural factors [4]. For example, qualitative studies of persons’ experiences with health and illness are more amenable to studying the impact of gender, while clinical trials of drug therapies are more amenable to study the impact of sex. To accommodate these possibilities, we used the term “sex/gender”–not to imply that the two concepts are synonymous, but to serve as shorthand for “and/or” throughout the metrics.
- Third, not all elements of the metrics will apply to all types of research studies, nor all study designs. For example, some of the assessment categories will not be applicable to qualitative studies (such as ensuring sufficient sample size for powering statistical analyses).
- Finally, the metrics are not only applicable to studies that investigate sex/gender *differences*, but are equally useful in study proposals focused on *one* sex or gender. Understanding the role of these factors in shaping health experiences and outcomes is also important for revealing within-group differences in sex- or gender-specific studies [36].

### Essential metrics for assessing sex & gender integration in health research proposals involving human participants

The final version of the metrics is presented in Table 1.

**Table 1:** Essential Metrics for Assessing Sex & Gender Integration in Health Research Proposals Involving Human Participants

Proposal Section	Questions to Ask	Examples	Assessment Scale
**1. Literature Review & Research Objectives**	• Does the literature review include consideration of sex/gender? • Do the research objectives include exploration of sex/gender?	• Are knowledge, gaps or questions about sex/gender raised in the literature review? • Are gaps or questions about sex/gender addressed in the research objectives?	• **Excellent:** substantial consideration of sex/gender throughout the literature review; explicit and thorough exploration of sex/gender identified in research objectives. • **Good:** some consideration of sex/gender in literature review and research objectives, but with some potential to be expanded. • **Fair:** minimal consideration of sex/gender in literature review and research objectives, numerous critical gaps remain. • **Poor:** sex/gender not considered at all in the literature review and research objectives.
**2. Research Design, Methods, & Analysis Plan**	**a) Population** • Has sex/gender been considered in the inclusion and exclusion criteria? • Has sex/gender been considered in the calculation of sample size?	• Are some populations inappropriately excluded on the basis of sex/gender by the inclusion/exclusion criteria? • To what extent has justification been provided for the inclusion/exclusion of populations based on sex/gender? • Has the sample size been sufficiently powered to identify potentially relevant sex/gender findings?	• **Excellent:** substantial justification provided for sex/gender related inclusion/exclusion criteria. • **Good:** clear justification provided for sex/gender related inclusion/exclusion criteria, but with some potential to be expanded. • **Fair:** minimal justification provided for sex/gender related inclusion/exclusion criteria, numerous critical gaps remain. • **Poor:** no justification for sex/gender related inclusion/exclusion criteria. • Sufficient sample size to allow for potential sex/gender findings: □ Yes □ No
	**b) Participant Recruitment & Retention:** • Has sex/gender been considered in the recruitment and retention strategies to ensure as broad as possible study participation?	• Are there potential sex/gender related barriers to participation for some populations who should be included? Consider the accompanying recruitment materials; for example, where will recruitment posters be located, and who is represented on the posters? • Are there potential sex/gender related barriers to retention? For example, will child care be available or travel costs offset for participants? Is consideration given to sex-specific dosing strategies to prevent adverse events that may be common in males/females?	• **Excellent:** consideration of sex/gender in recruitment and retention strategies will ensure the broadest possible participation by study population(s). • **Good:** some consideration given to sex/gender in the recruitment and retention strategies, but there remains some potential to further broaden participation. • **Fair:** minimal inclusion of sex/gender in the recruitment and retention strategies may result in numerous populations being inappropriately excluded. • **Poor:** sex/gender not considered in recruitment and retention strategies. • **Not Applicable:** No recruitment needed.
	**c) Data Collection Tools:** • Do the data collection tools capture information relevant to sex/gender?	• Do the participant intake forms and other tools (e.g. questionnaires, interview guides) capture sex (e.g. male, female) and/or gender identities (e.g. man, woman, transgender, two-spirit, etc.)? • Will the tools used to collect data include variables to conduct analyses of the influence of sex/gender?	• **Excellent:** all tools reflect the widest possible range of sex/gender identities; tools will collect extensive data relevant to conducting sex/gender analyses. • **Good:** tools reflect a range of sex/gender identities and will collect some data relevant to sex/gender, but with potential to be expanded. • **Fair:** tools reflect minimal data on sex/gender identities and numerous critical gaps remain in the collection of sex/gender relevant data. • **Poor:** no sex/gender data will be collected.
	**d) Data Analysis Plan:** • Does the proposal include a plan to analyze the impact of sex/gender on study findings?	• Will key variables be analyzed and reported disaggregated by sex/gender? • In single-sex/gender studies: is there a plan to investigate differences within this population?	• **Excellent:** all data will be analyzed in relation to sex/gender. • **Good:** most data will be analyzed in relation to sex/gender, but there is some potential to further expand the sex/gender analyses. • **Fair:** minimal data will be analyzed in relation to sex/gender, numerous critical gaps remain. • **Poor:** no inclusion of sex/gender in the analysis plan.
**3. Knowledge Translation Plan**	• Has sex/gender been considered in the knowledge translation plan?	• Are knowledge translation strategies customized for relevance to a range of populations? For example, is there a plan to present findings relevant to specific participant populations based on sex/gender?	• **Excellent:** the knowledge translation plan explicitly notes sex/gender considerations and is tailored to the widest possible range of populations. • **Good:** the knowledge translation plan shows some consideration of sex/gender, but there is some potential to expand the possibilities for tailoring to a range of populations. • **Fair:** numerous critical gaps remain in the knowledge translation plan’s consideration of sex/gender and tailoring to a range of populations. • **Poor:** no consideration of sex/gender in the knowledge translation plan.

As indicated in the first column of Table 1, the metrics are divided into 3 primary sections reflecting the stages of research development: 1) Literature Review & Research Objectives, 2) Research Design, Methods, & Analysis Plan, and 3) Knowledge Translation Plan. For each of these three sections there is a question or series of subsection questions on sex/gender integration, examples to illustrate each question, and a descriptive assessment scale to evaluate the extent to which the proposal addresses the questions. Each of the three sections and the rationale for their inclusion are explored in detail below.

#### Section 1: literature review & research objectives

Questions asked: Does the literature review include consideration of sex/gender? Do the research objectives include exploration of sex/gender?

Examples: Are current knowledge, gaps or new questions about sex/gender raised in the literature review? Are gaps or new questions about sex/gender addressed in the research objectives?

Rationale: Inclusion of sex/gender in the literature review is essential to determining what is already known about the impact of these factors on the proposed topic of study [29]. Excellent inclusion of sex/gender in the review should not only outline what is presently known about the incidence, prevalence, risk factors, pathophysiology, treatment and experience of health and illness, but also gaps in our understanding and questions that might arise from these gaps [42]. The literature review should be used to inform the basis of an explicit exploration of sex/gender in the proposed research objectives.

#### Section 2: research design, methods, & analysis plan

a) Population

Questions asked: Has sex/gender been considered in the inclusion and exclusion criteria? Has sex/gender been considered in the calculation of sample size?

Examples: Are some populations inappropriately excluded on the basis of sex/gender by the inclusion/exclusion criteria? To what extent has justification been provided for the inclusion/exclusion of populations based on sex/gender? Has the sample size been sufficiently powered to identify potentially relevant sex/gender findings?

Rationale: The proposal should identify and thoroughly justify any inclusion and/or exclusion criteria based on sex/gender; consideration should be given to whether some populations have been inappropriately excluded on the basis of sex/gender considerations [23]. Additionally, other factors that intersect with sex and/or gender that are of relevance to the research question should be noted. For example, if the inclusion criteria exclude populations based on age cut-offs, consider potential sex-based differences in the age of onset for the condition under study [43]. The proposed sample size should be sufficient to permit analyses illuminating sex/gender findings; in the case of quantitative research, this will require consideration of whether the sample size is sufficiently powered, while in qualitative research the sample size needs to be large enough to capture a range of experiences among participants. Note that the question of sufficient sample size is the only metric for which it is not possible to assess relative quality; it is either sufficient or not sufficient.

b) Participant Recruitment & Retention (if applicable)

Questions asked: Has sex/gender been considered in the recruitment and retention strategies to ensure as broad as possible study participation?

Examples: Are there potential sex/gender related barriers to participation for some populations who should be included? Consider the accompanying recruitment materials; for example, where will recruitment posters be located, and who is represented on the posters? In terms of retention, will child care be available or travel costs offset for participants? Is consideration given to sex-specific dosing strategies to prevent adverse events that may be common in males/females?

Rationale: The proposed study recruitment and retention strategies should outline possible sex-/gender-based barriers and provide a clearly articulated plan to ensure the broadest possible range of participation. For example, gendered differences in help-seeking and health care utilization [44] may impact opportunities for recruiting men into the study sample. Similarly, the gendered division of labour in the home [45] may warrant offering childcare or flexible participation hours to recruit and retain women participants. This section will not be relevant for studies that do not involve recruitment of human subjects, which should be evaluated as “not applicable” in the accompanying assessment scale.

c) Data Collection Tools

Questions asked: Do the data collection tools capture information relevant to sex/gender?

Examples: Do the participant intake forms and other tools (e.g. questionnaires, interview guides) capture sex (e.g. male, female) and/or gender identities (e.g. man, woman, transgender, two-spirit, etc.)? Will the tools used to collect data include variables to conduct analyses of the influence of sex/gender?

Rationale: Data collection tools such as surveys, focus group questionnaires, patient intake and demographic forms should be designed to capture an array of relevant sex/gender information, allowing for robust analyses of the influence of sex/gender on study outcomes [37]. Additionally, researchers are urged to move beyond the sex and gender binary of male/female and man/woman in designing data collection tools. These categories do not the capture the full range of possibilities in human expressions of sex and gender, which includes intersex and transgender individuals [46, 47].

d) Data Analysis Plan

Questions asked: Does the proposal include a plan to analyze the impact of sex/gender on study findings?

Examples: Will key variables be analyzed and reported disaggregated by sex/gender? In single-sex/gender studies: is there a plan to investigate differences within this population?

Rationale: The proposal should include an explicit plan to conduct sex/gender analyses, ideally by disaggregating and analyzing all research results by sex/gender [29]. Controlling for sex/gender means that valuable information may be lost regarding whether and how the study outcomes differ by sex/gender [4]. The proposal should thus clearly articulate how the study’s analyses will investigate the potential *impact* of sex/gender on study findings [23]. In studies specific to only one sex or gender, there should be a clearly articulated plan to analyze and report differences among the study population [36].

#### Section 3: knowledge translation plan

Questions asked: Has sex/gender been considered in the knowledge translation plan?

Examples: Are knowledge translation strategies customized for relevance to a range of populations? For example, is there a plan to present findings relevant to specific participant populations based on sex/gender?

Rationale: The knowledge translation plan should reflect sex/gender considerations in strategies for disseminating the research findings [37]. For example, a proposal with an excellent knowledge translation plan would articulate a clear plan for selecting and tailoring interventions based on sex/gender, accounting for potential sex/gender barriers and facilitators to the knowledge translation process [48].

## Discussion

The *Essential Metrics for Assessing Sex & Gender Integration in Health Research Proposals Involving Human Participants* are among the first to provide a comprehensive guide for assessing the quality of sex and gender integration in health research proposals. The metrics are designed for use by researchers preparing proposals and by reviewers evaluating proposals for funding opportunities. Funding agencies require strategies for encouraging meaningful and thorough consideration of sex and gender in grant applications [49]. Our metrics are one such mechanism, providing criteria for proposal reviewers on what constitutes high-quality integration. Further, as evidenced by the inconsistent and uneven integration of sex and gender across the fields of health research [5–9], researchers developing a proposal require criteria for ‘best practices’ in sex and gender integration. While we recognize that these metrics do not necessarily resolve all of the challenges of sex and gender inclusion [50, 51], they do provide a practical strategy for helping researchers and reviewers think beyond a tokenistic or ‘checkbox’ approach. The metrics also complement funding agencies’ efforts to ensure that reviewers have sufficient instruction in and understanding of sex and gender integration when evaluating applications [38].

Health researchers have been challenged to find ways to reduce research “waste” through better consideration of what evidence is already known, increasing the yield of research evidence, and enhancing the applicability of their research[52]. Identifying opportunities to include sex and gender at the proposal stage can help reduce potentially avoidable research waste by both building our knowledge of how sex and gender impact health, as well as enhancing evidence to inform health practice and policy. There is substantial evidence to show that prioritizing the integration of sex and gender–and considering these factors throughout the research process–leads to innovative evidence to improve the health of all [12]. For example, studying the influence of sex and gender on pharmacokinetics has advanced our understanding of differences in drug tolerability and efficacy, helping to inform safer and more effective prescribing [53]. Sex- and gender-based disparities have been noted in the manifestation and management of cardiovascular disease, revealing crucial opportunities to enhance treatment [54]. Investigating how sex and gender inform risk and protective factors in dementia and Alzheimer’s disease has also helped to establish recommendations for innovative future research [55, 56]. Use of the metrics may help ensure similarly promising lines of inquiry are pursued in all fields of health research.

The metrics address the limitations we have identified in the few existing decision-making aides for reviewing proposals [15–18], providing benchmarks for excellence in sex and gender integration as well as an assessment scale for evaluating quality. To the best of our knowledge the closest comparable evaluation strategy is offered by Tomás et al.’s questionnaire, which allow reviewers to order proposals into three “levels” of integration: “difference by sex” (basic research that disaggregates findings by sex), “gender sensitive” (research that looks at the impact of gender differences on health), and “feminist research” (research that addresses health inequalities). These are more accurately described as categories or types of research rather than levels of integration, given that whether a proposed study will examine sex, gender, or both depends on the purpose of the study and whether biological or psychosocial/sociocultural factors or both are relevant to that purpose [4]. In contrast, our metrics are broadly applicable to evaluating integration quality within each phase of the research process and across a broad range of study designs, regardless of study purpose.

A strength of the metrics is that they have been developed on the basis of published resources for conducting sex and gender integration, as well as with input from international experts in sex- and gender-based health research. A potential limitation is that, as our reviewers of the metrics were sex and gender health research experts, their responses to our review and feedback survey may have been biased towards reporting the metrics as useful, clear and complete. To address this potential limitation we have taken steps to assist non-expert users, including providing contextualizing instructions for appropriate use of the metrics as well as examples to illustrate the kinds of factors that should be considered in evaluating each proposal component. Additionally, limiting the assessment scale to four clearly differentiated and descriptive levels of quality is intended to help all reviewers develop consistency in their evaluation of proposals. We will continue to update the metrics as new information becomes available and the metrics are applied by different groups in practice.

## Conclusions

The *Essential Metrics for Assessing Sex & Gender Integration in Health Research Proposals Involving Human Participants* is a scaled assessment tool that can be used by both reviewers and researchers to evaluate the quality of sex and gender integration in any health research proposal. The metrics support growing efforts to promote incorporation of sex and gender as a means of enhancing the value, applicability and quality of health research. By enhancing the integration of sex and gender in proposals, use of the metrics will help to foster high-quality, meaningful integration of sex and gender throughout the research process, resulting in better quality evidence to inform health care for all.
